# Microbiological quality analysis of inoculants based on *Bradyrhizobium* spp. and *Azospirillum brasilense* produced “on farm” reveals high contamination with non-target microorganisms

**DOI:** 10.1007/s42770-021-00649-2

**Published:** 2022-01-01

**Authors:** Camila Rafaeli Bocatti, Eduara Ferreira, Renan Augusto Ribeiro, Ligia Maria de Oliveira Chueire, Jakeline Renata Marçon Delamuta, Renata Katsuko Takayama Kobayashi, Mariangela Hungria, Marco Antonio Nogueira

**Affiliations:** 1 Department of Microbiology, Universidade Estadual de Londrina, C. Postal 10.011, Londrina, PR 86057-970 Brazil; 2 Embrapa Soja, C. Postal 4006, Londrina, PR 86081-981 Brazil; 3 Conselho Nacional de Desenvolvimento Científico e Tecnológico (CNPq), Brasília, DF Brazil

**Keywords:** Inoculation, Biological nitrogen fixation, Plant growth–promoting bacteria, Pathogenic microorganisms, On farm fermentation

## Abstract

The use of inoculants carrying diazotrophic and other plant growth–promoting bacteria plays an essential role in the Brazilian agriculture, with a growing use of microorganism-based bioproducts. However, in the last few years, some farmers have multiplied microorganisms in the farm, known as “on farm” production, including inoculants of *Bradyrhizobium* spp. for soybean (*Glycine max* L. Merrill.) and *Azospirillum brasilense* for corn (*Zea mays* L.) or co-inoculation in soybean. The objective was to assess the microbiological quality of such inoculants concerning the target microorganisms and contaminants. In the laboratory, 18 samples taken in five states were serial diluted and spread on culture media for obtaining pure and morphologically distinct colonies of bacteria, totaling 85 isolates. Molecular analysis based on partial sequencing of the 16S rRNA gene revealed 25 genera of which 44% harbor species potentially pathogenic to humans; only one of the isolates was identified as *Azospirillum brasilense*, whereas no isolate was identified as *Bradyrhizobium*. Among 34 isolates belonging to genera harboring species potentially pathogenic to humans, 12 had no resistance to antibiotics, six presented intrinsic resistance, and 18 presented non-intrinsic resistance to at least one antibiotic. One of the samples analyzed with a shotgun-based metagenomics approach to check for the microbial diversity showed several genera of microorganisms, mainly *Acetobacter* (~ 32% of sequences) but not the target microorganism. The samples of inoculants produced on farm were highly contaminated with non-target microorganisms, some of them carrying multiple resistances to antibiotics.

## Introduction

Soybean (*Glycine max* L. Merr.) and corn (*Zea mays* L.) are the main Brazilian grain crops [1], with a production ~ 125 million tons in ~ 37 million hectares of soybean, and ~ 102.5 million tons in ~ 18.5 million hectares of corn [2]. The symbiosis between soybean and elite *Bradyrhizobium* strains can supply the most part of the required N via biological nitrogen fixation (BNF) [3] and grain yield increases by 8% due to inoculation [4]. In corn, yield increase due to inoculation with *Azospirillum brasilense* has been attributed to bacterial phytohormones [5, 6]. Co-inoculation of soybean with *Bradyrhizobium* spp. and *A. brasilense* has doubled the benefits compared with single inoculation [7, 8].

Brazil has a long tradition in research with inoculants containing rhizobia and *Azospirillum*, and legislation for quality control of inoculants. According to the standards established by the Ministry of Agriculture, Livestock and Food Supply (MAPA), commercial inoculants must have the minimal concentration of 10^9^ viable cells of *Bradyrhizobium* and 10^8^ cells of *Azospirillum* per gram or milliliter of inoculant, no contaminants at the 10^−5^ dilution, and must carry only elite strains with recognized agronomic efficiency [9, 10].

The industrial production of inoculants is a complex process, but improvements in the last two decades have resulted in high-quality products in terms of cell concentrations, no contaminants, and very low cost, probably the cheapest inoculant in the world [11]. However, in the last five years, some farmers have tried to produce their own bioproducts, including inoculants in the farm, using simplified biofactories, known as “on farm” production. In most cases, the production system is rudimentary and varies in terms of installations, equipment, microbiological standards, and technical capacity. Very often the bioproducts are produced in fermenters, open tanks, or even water tanks, without appropriate control of contaminations, which may result in highly contaminated, non-effective products [12, 13].

The objective of this study was to assess the microbiological quality of inoculants based on *Bradyrhizobium* spp. and *A. brasilense* produced on farm in Brazil, concerning the intended microorganisms, presence, and characterization of probable contaminants.

## Materials and methods

### Sampling

Sampling and transportation kits containing Styrofoam box, sterile 50-mL Falcon-type conical tubes, sterile 30-mL disposable syringes, disposable gloves, Parafilm M® for sealing the tubes, and cooling packs were sent to farmers interested to know the microbiological quality of their inoculants produced on farm. The kit included a protocol for sampling, emphasizing aseptic procedures and an identification form. Immediately after sampling, two aliquots per tank or fermenter were packed with cooling packs in the Styrofoam box and sent back by express postal service or personally delivered in the Laboratory for Soil Biotechnology at Embrapa Soja. A total of 18 samples were obtained during 2019/20 cropping season, six aiming *Bradyrhizobium* and 12 aiming *Azospirillum* as target microorganisms (Table 1). These samples were obtained from five states: São Paulo (six), Bahia (two), Paraná (five), Rio Grande do Sul (three), and Mato Grosso (two). For comparative purposes, commercial inoculants containing *A. brasilense* strains Ab-V5 and Ab-V6 (C1, lot 1,108,718), *B. diazoefficiens* strain SEMIA 5080 and *B. japonicum* strain SEMIA 5079 (C2, lot 0,135,218), and *Bradyrhizobium elkanii* strains SEMIA 587 and SEMIA 5019 (C3, lot 19,014,223) were included. It is worth mentioning that, although not mandatory, commercial inoculants in Brazil usually contain two bacterial strains.

**Table 1:** Origin of the sample (municipality and state), target microorganism, pH, electrical conductivity, odor, type of multiplication, and growth time during the sampling of inoculants produced on farm in the 2019/20 growth season

Sample	Municipality-State	Target microorganism	pH	Electrical Conductivity (μS/cm)	Odor	Type of multiplication	Growth time
1	Presidente Bernardes-SP	*Bradyrhizobium*	5.7	4000	Putrid	Open tanks	2 days
2	Presidente Bernardes-SP	*Azospirillum*	4.0	2900	Sour	Open tanks	1 day
3	Barreiras-BA	*Bradyrhizobium*	4.9	2100	Sour	Fermenter	10 days
4	Marilândia do Sul-PR	*Azospirillum*	4.4	890	Sour	Open tanks	2 days
5	Mauá da Serra-PR	*Azospirillum*	5.9	800	Sour	Open tanks	4 h
6	Mauá da Serra-PR	*Azospirillum*	3.6	1030	Sour	Open tanks	7 days
7	Luís Eduardo Magalhães-BA	*Azospirillum*	7.2	2060	Fecal	Open tanks	5 days
8	Panambi-RS	*Azospirillum*	3.9	1620	Urine	Open tanks	1 day
9	Palotina-PR	*Bradyrhizobium*	5.3	6890	Sour	Open tanks	2 days
10	Palotina-PR	*Azospirillum*	5.0	8390	Garbage	Open tanks	2 days
11	Sorriso-MT	*Azospirillum*	3.9	5930	Sour	Open tanks	3 days
12	Sorriso-MT	*Azospirillum*	4.4	4640	Fecal	Open tanks	3 days
13	Panambi-RS	*Bradyrhizobium*	4.7	1870	Yeast	Fermenter	2 days
14	Panambi-RS	*Azospirillum*	4.8	2200	Yeast	Fermenter	1 day
15	Salto Grande-SP	*Bradyrhizobium*	4.0	3830	Sour	Open tanks	3 days
16	Salto Grande-SP	*Azospirillum*	5.5	7020	Fecal	Open tanks	1 day
17	Lutécia-SP	*Bradyrhizobium*	5.5	2760	Fecal	Not informed	Not informed
18	Lutécia-SP	*Azospirillum*	5.1	2910	Sour	Not informed	Not informed
C1	–	*Azospirillum*	7.1	9810	Vinegar	Industrial fermenter	–
C2	–	*Bradyrhizobium* spp.	7.1	1960	Yeast	Industrial fermenter	–
C3	–	*B. elkanii*	7.2	2200	Yeast	Industrial fermenter	–

### Physical–chemical and organoleptic properties

The samples and the commercial inoculants were evaluated for pH using a pH-meter model FiveEasy Plus pH-meter FP20 (METTLER TOLEDO, Ohio, USA) and electrical conductivity in a digital conductivity-meter Tec-4MP (TECNAL, Piracicaba, Brazil). A sensorial analysis was based on the “odor wheel” described by McGinley and McGinley [14], which highlights eight categories of odors.

### Isolation of morphotypes

Under aseptic conditions, serial dilutions were made in sterile 0.85% NaCl saline and 100-μL aliquots of the 10^−5^, 10^−6^, and 10^−7^ dilutions were spread on five different culture media: modified YMA (Yeast Mannitol Agar) for *Bradyrhizobium* [15]; RC (Rojo Congo) [16] for *Azospirillum*; LB (Luria Bertani) [17]; NA (Nutrient Agar) [18]; and Sabouraud [19]. The different culture media aimed to check for occurrence of typical colonies of the target microorganisms, and increase the chance of obtaining as many as possible contaminating isolates able to grow in these culture media.

After spreading on each medium, plates were incubated at 28 ± 1 °C in the inverted position in a growth room and were daily observed for 7 days. The morphologically distinct colonies in each culture medium were streaked again on the same culture medium to select single colonies. To avoid morphologically distinct isolates due to the growth medium, all isolates were streaked on NA to standardize the morphology of colonies. Finally, morphologically distinct isolates in NA medium were cryopreserved in NA broth with 30% glycerol at − 80 °C for further analysis.

Prior to cryopreservation, all isolates were observed at 400 × magnification under an optical microscope (AxioLab A1, Zeiss) coupled to an AxioCam ERc 5 s digital video camera system (Zeiss) for recognition of typical yeast traits such as nucleus, vacuole, and cell dimensions. Isolates identified as yeasts were not submitted to further analysis.

### Molecular identification of isolates

Total DNA of morphologically distinct isolates was extracted with the DNeasy Blood and Tissue Kit (Qiagen), according to the manufacturer’s instructions. After extraction, the integrity of DNA was verified by electrophoresis in 1% agarose gel. The 16S rRNA gene was amplified as described [20] with universal primers fD1 (5′-AGAGTTTGATCCTGGCTCAG-3′) and rD1(5′-AAGGAGGTGATCCAGCC-3′) for phylogenetic studies of bacteria, flanking nearly the entire region of the 16S rRNA gene (~ 1,500 bp) [21]. The PCR products were purified with the PureLink™ Quick PCR Purification Kit (Invitrogen), according to the manufacturer’s instructions. Sequencing was performed in an ABI3500xL analyzer (Applied Biosystems) as described [22]. Fragment sequences ranging from 484 to 1139 bp were analyzed using the software Bionumerics version 7.6 and identification was based on comparison with the NCBI GenBank database using the BLAST tool for nucleotides (https://blast.ncbi.nlm.nih.gov/Blast.cgi).

### Metagenome analysis

To have a broader view of the diversity of microorganisms that might not have grown on the culture media, or occurring at low concentrations in the sample, metagenomic analysis was performed in sample 10, from Palotina, PR. We used the shotgun approach, sequencing all DNA fragments extracted from the sample, without previous amplification of any specific region, as described before [23]. The shotgun approach detects higher diversity in a sample as well as microorganisms in all domains of life and, if required, can also be used for functional analysis. For the metagenomics analysis, total DNA was extracted with the DNeasy blood and tissue kit (Qiagen) and used to build the library with the Nextera XT kit, according to the manufacturer’s procedure. The library was processed on the MiSeq platform (Illumina) at Embrapa Soja, and the sequences were assembled with the A5-miseq pipeline (de novo) version 20,140,604. The sequenced fragments were uploaded to the MG-RAST v.4.0.4 (RAST—http://metagenomics.anl.gov) and submitted to automatic annotation in the server based on the NCBI BLAST and SEED databases [24].

### Susceptibility to antimicrobials

After molecular identification, isolates belonging to potentially pathogenic genera were subjected to evaluation of susceptibility to antimicrobials by the Disk-Diffusion Test [25]. Cells grown for 24–48 h on NA medium were suspended in sterile saline (0.85% NaCl) until a turbidity compatible with the McFarland scale 0.5 (~ 1.5 × 10^8^ CFU mL^−1^). The suspension was then inoculated on the Müeller-Hinton [26] agar plate using a sterile swab. Then, paper disks impregnated with antimicrobials were added, as indicated in the annual updates of the Clinical and Laboratory Standards Institute (CLSI) [27].

The antimicrobials and their concentrations per disk were as follows: amikacin 30 μg, amoxicillin + clavulanate 20/10 μg, ampicillin 10 μg, ampicillin + sulbactam 10/10 μg, aztreonam 30 μg, cefazolin 30 μg, cefepime 30 μg, cefotaxime 30 μg, cefoxitin 30 μg, ceftazidime 30 μg, ceftriaxone 30 μg, ciprofloxacin 5 μg, clindamycin 2 μg, chlorampheniol 30 μg, erythromycin 15 μg, ertapenem 10 μg, gentamicin 10 μg (120 μg for *Enterococcus faecalis*), imipenem 10 μg, linezolid 30 μg, levofloxacin 5 μg, meropenem 10 μg, penicillin 10 μg, piperacillin + tazobactam 100/10 μg, streptomycin 10 μg (300 μg for *E. faecalis*), sulbactam 10 μg, sulfamethoxazole + trimethoprim 1.25/23.75 μg, tetracycline 30 μg, and vancomycin 30 μg. The plates were incubated at 36 °C and the patterns of inhibition halos around each disk were evaluated after 18–24 h, as indicated by CLSI [27].

## Results

### Physical–chemical and organoleptic properties

The physical–chemical and organoleptic properties, type of equipment used for multiplication (open tanks or fermenters), and growth time (from inoculation up to sampling) of the 18 samples are shown in Table 1. The pH ranged from 3.6 (sample 6) to 7.2 (sample 7), the latter was the only with slightly alkaline pH, whereas the others were acidic, below pH 6.0. The electrical conductivity ranged from 800 (sample 5) to 8390 μS cm^−1^ (sample 10). Among the commercial inoculants, pH was slightly alkaline and the one containing *A. brasilense* presented the highest electrical conductivity. The cell concentration in the commercial inoculant C1 (*A. brasilense* Ab-V5 and Ab-V6) was 1.01 × 10^9^ CFU mL^−1^; in C2 (*Bradyrhizobium* spp. SEMIA 5079 and SEMIA 5080) was 6.30 × 10^9^ CFU mL^−1^; and in C3 (*B. elkanii* SEMIA 587 and SEMIA 5019) was 8.47 × 10^9^ CFU mL^−1^. No contaminants were found in the commercial inoculants.

In the sensorial analysis [14], only two samples were classified as “yeast” (samples 13 and 14), whereas the others presented odors classified as “offensive,” which might be attributed to putrefaction processes. The commercial inoculants, however, presented odors classified as “vinegar” and “yeast” for *Azospirillum* and *Bradyrhizobium*, respectively (Table 1). Among 18 samples, three were declared as multiplied in fermenters, 13 in open tanks, and two were not informed. The growth time ranged from 4 h (sample 5) to 10 days (sample 3).

### Bacterial isolation and molecular identification

**Fig. 1:**
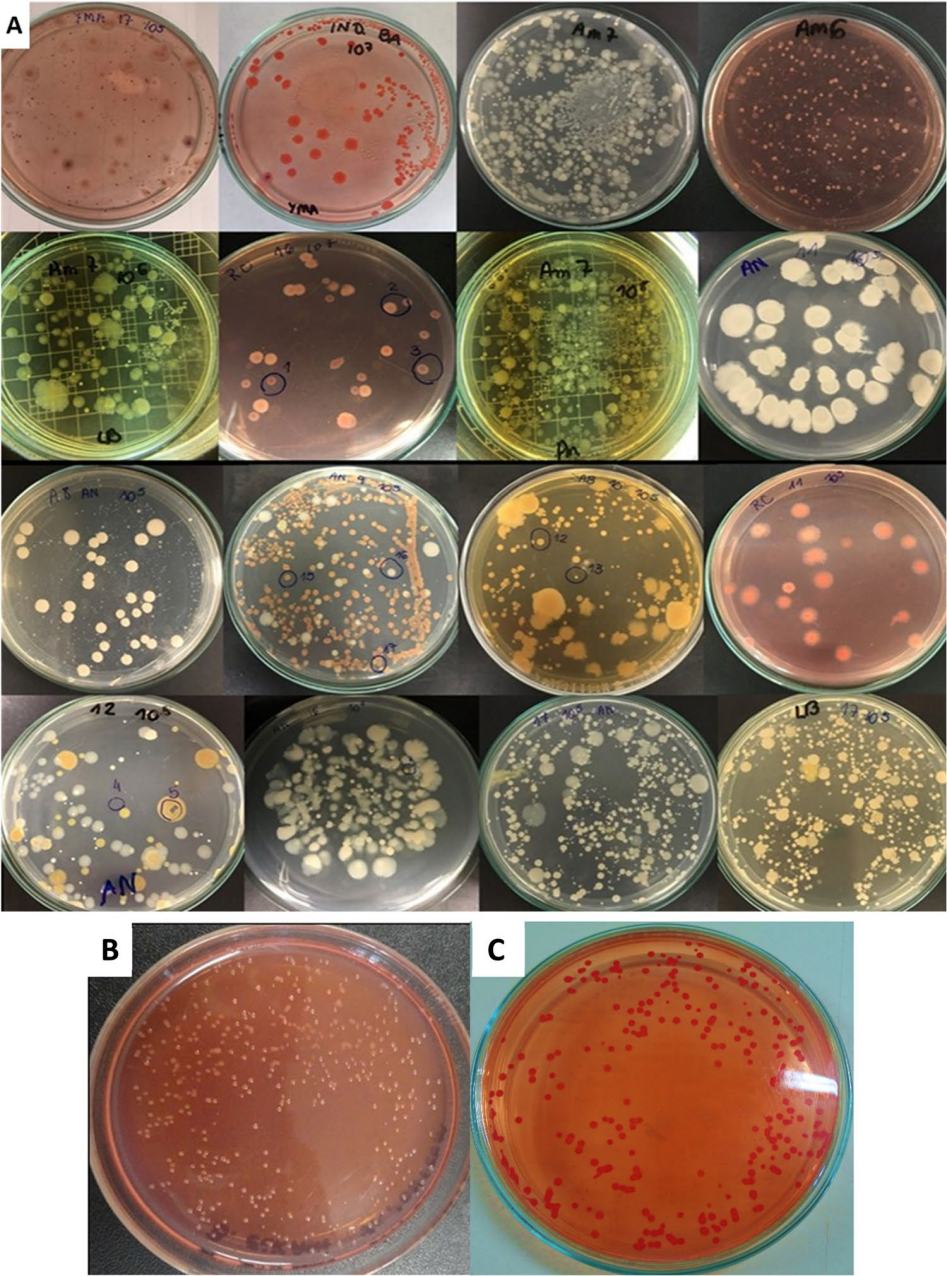
**A** Overview of Petri’s dishes containing different culture media inoculated with samples of inoculants produced on farm in the 2019/2020 growth season aiming the multiplication of *Bradyrhizobium* spp. or *Azospirillum brasilense*. Petri’s dishes containing pure colonies of *Bradyrhizobium* (**B**) and *Azospirillum* (**C**), grown on YMA (Yeast Mannitol Agar) and RC (Rojo Congo) culture media, respectively

The plating for isolation in culture media indicated a variety of colony morphotypes, as exemplified in Fig. 1A, suggesting occurrence of contaminants, as they differed from typical colonies of *Bradyrhizobium* (Fig. 1B) and *Azospirillum* (Fig. 1C).

A total of 84 morphologically distinct isolates were obtained from the 18 samples (Table 2). Sequencing of 16S rRNA gene resulted in sequences that ranged from 484 to 1140 bp, most of them above 1000 bp. Comparisons of sequences in the GenBank showed 44 isolates with similarity ≥ 99% and 28 between 99 and 97.2% with deposited sequences, and coverage between 95 and 100%. Finally, 12 isolates were identified as yeasts based on the cell morphology (size, presence of nucleus, and budding) and were not sequenced.

**Table 2:** Similarity based on partial sequencing of the 16S rRNA gene of bacterial isolates obtained from samples of inoculants produced on farm, and commercial inoculants, in the 2019/20 growth season aiming the multiplication of *Bradyrhizobium* spp. and *Azospirillum brasilense*, and their potential as human pathogens

Sample	DNA fragment (bp)*	Likely species/genus	Cover, %*	Identity, %*	GenBank access number	Potentially human pathogen	Reference
1.1 ***	1139	*Citrobacter braakii*	99	99.56	LR134214.1	Yes	Hirai et al. [28]
1.2	1139	*Enterobacter bugandensis*	99	99.68	CP039453.1	Yes	Pati et al. [29]
1.3	1134	*Acinetobacter baumannii*	99	100	CP044356.1	Yes	McConnell et al. [30]
1.4	1134	*Rummeliibacillus pycnus*	100	100	JF833091.2	No	Her; Kim [31]
1.5	1058	*Enterococcus faecalis*	98	100	CP041738.1	Yes	Poulsen et al. [32]
2.1	1134	*Bacillus megaterium*	99	99.82	CP032527.2	No	Faccin et al. [33]
2.2	1139	*Citrobacter* sp*.*	99	98.91	MN521452.1	Depends on the species	Brenner et al. [34] Hasan; Sultana; Hossain [35]
2.3	1139	*Escherichia coli*	99	99.82	CP044314.1	Yes	Forson et al. [36]
2.4	1009	*Enterococcus faecalis*	100	100	MN420846.1	Yes	Poulsen et al. [32]
2.5	1110	*Lactococcus lactis*	99	99.82	MN466963.1	No	Guerra [37]
2.6	1140	*Kurthia gibsonii*	95	100	KJ872770.1	No	Dworkin et al. [38]
3.1	1097	*Acetobacter syzygii*	99	99.72	NR_113850.1	No	Aghazadeh; Pouralibaba; Yari Khosroushahi [39]
3.2	1127	*Lactobacillus farraginis*	98	100	NR_041467.1	No	Endo; Okada [40]
3.3	1136	*Lactobacillus rhamnosus*	99	100	AB008211.1	No	Jung et al. [41]
3.4	1127	*Enterococcus* sp*.*	99	99.73	AJ132470.1	Depends on the species	Camargo et al. [42]
4.1	1134	*Lactococcus lactis*	100	99.82	AM944595.1	No	Guerra [37]
4.2	1132	*Acinetobacter nosocomialis*	99	100	CP042994.1	Yes	Knight et al. [43]
4.3	–	Yeast**	–	–	–	Depends on the species	Moyad [44] Hafed et al. [45]
4.4	812	*Enterobacter* sp*.*	100	99.88	MK999972.1	Yes	Kus; Burrows [46]
4.5	–	Yeast**	–	–	–	Depends on the species	Moyad [44] Hafed et al. [45]
4.6	1052	*Raoultella* sp*.*	99	99.62	CP026047.1	Rarely	Ramirez-Quintelo; Chavarriaga-Restrepo [47]
4.7	1134	*Stenotrophomonas maltophilia*	100	99.91	CP028899.1	Yes	Kasper et al. [48]
4.8	1081	*Acinetobacter* sp*.*	100	99.91	MN443626.1	Depends on the species	Chagas [49]
5.1	1132	*Lactococcus lactis*	100	100	AM944595.1	No	Guerra [37]
5.2	1069	*Azospirillum brasilense*	99	100	CP033320.1	No	Santini et al. [50]
5.3	1138	*Exiguobacterium acetylicum*	99	99.47	CP030931.1	No	Selvakumar et al. [51]
6.1	1120	*Bacillus subtilis*	100	100	CP035164.1	No	Van Dijl; Hecker [52]
6.2	1132	*Lactococcus lactis*	100	99.86	AM944595.1	No	Guerra [37]
7.1	1110	*Citrobacter* sp*.*	100	99.18	CP021963.1	Depends on the species	Brenner et al. [34] Hasan; Sultana; Hossain [35]
7.2	1089	*Comamonas aquatica*	100	99.91	CP016603.1	No	Dai et al. [53]
7.3	1058	*Klebsiella pneumoniae*	98	99.62	AB641122.1	Yes	Boszczowski et al. [54]
7.4	1100	*Stenotrophomonas* sp*.*	99	99.91	LS483406.1	Only *S. maltophilia*	Kasper et al. [48]
7.5	1084	*Atlantibacter hermannii*	98	99.91	CP042941.1	Yes	Ioannou [55]
8.1	1104	*Lactococcus lactis*	100	99.37	CP043523.1	No	Guerra [37]
8.2	–	Yeast **	–	–	–	Depends on the species	Moyad [44] Hafed et al. [45]
8.3	1039	*Lactobacillus* sp*.*	100	97.02	LC438378.1	No	Delgado et al. [56]
8.4	1081	*Enterococcus faecalis*	100	99.08	CP045918.1	Yes	Poulsen et al. [32]
8.5	1083	*Burkholderia contaminans*	99	99.25	MW195002.1	Yes	Power et al. [57]
9.1	–	Yeast **	–	–	–	Depends on the species	Moyad [44] Hafed et al. [45]
9.2	1058	*Acetobacter* sp*.*	99	99.34	LN609302.1	No	Kommanee et al. [58]
9.3	1107	*Enterococcus* sp*.*	100	98.65	AJ626904.1	Depends on the species	Camargo et al. [42]
9.4	1129	*Lactococcus lactis*	100	99.67	AM944595.1	No	Guerra [37]
9.5	1138	*Bacillus subtilis*	99	99.59	MN415973.1	No	Van Dijl; Hecker [52]
9.6	1074	*Kocuria* sp*.*	99	98.32	AM179882.1	Depends on the species	Kandi et al. [59]
9.7	1062	*Terribacillus goriensis*	99	99.10	DQ519571.1	No	Krishnamurthi; Chakrabarti [60]
9.8	–	Yeast **	–	–	–	Depends on the species	Moyad [44] Hafed et al. [45]
9.9	1119	*Lactobacillus* sp*.*	100	98.21	NR_028658.1	No	Delgado et al. [56]
10.1	1133	*Enterococcus faecalis*	99	99.76	CP045918.1	Yes	Poulsen et al. [32]
10.2	1045	*Acetobacter* sp*.*	98	99.33	LN609302.1	No	Kommanee et al. [58]
10.3	1076	*Lactobacillus* sp*.*	99	99.81	NR_028658.1	No	Delgado et al. [56]
10.4	–	Yeast **	–	–	–	Depends on the species	Moyad [44] Hafed et al. [45]
11.1	706	*Bacillus* sp*.*	100	98.45	GQ181150.1	Depends on the species	Tuazon et al. [61] Amin; Rakhisi; Ahmady [62]
11.2	637	*Paenibacillus* sp*.*	100	98.90	MW555628.1	Depends on the species	Sáez-Nieto et al. [63]
11.3	919	*Enterococcus hirae*	100	99.59	MN420858.1	Rarely	Bourafa et al. [64]
11.4	–	Yeast **	–	–	–	Depends on the species	Moyad [44] Hafed et al. [45]
11.5	1064	*Rummeliibacillus* sp*.*	99	98.85	MT512031.1	No	Her; Kim [31]
12.1	582	*Acinetobacter* sp*.*	100	98.31	MK210236.1	Depends on the species	Chagas [49]
12.2	–	Yeast **	–	–	–	Depends on the species	Moyad [44] Hafed et al. [45]
12.3	1018	*Burkholderia vietnamiensis*	100	99.21	MH547402.1	Yes	Ieranò et al. [65]
13.1	1081	*Lactococcus lactis*	100	99.72	AM944595.1	No	Guerra [37]
13.2	–	Yeast **	–	–	–	Depends on the species	Moyad [44] Hafed et al. [45]
13.3	1072	*Gluconobacter japonicus*	100	99.12	AB253433.1	No	Cañete-Rodríguez et al. [66]
13.4	975	*Acetobacter* sp*.*	100	98.87	MW261886.1	No	Kommanee et al. [58]
14.1	1094	*Enterococcus faecalis*	99	99.45	CP041738.1	Yes	Poulsen et al. [31]
14.2	–	Yeast **	–	–	–	Depends on the species	Moyad [44] Hafed et al. [45]
14.3	1050	*Weissella paramesenteroides*	100	99.60	AY342336.1	No	Libonatti et al. [67]
15.1	1021	*Lactobacillus rhamnosus*	100	98.53	CP044228.1	No	Jung et al. [41]
15.2	1046	*Staphylococcus epidermidis*	100	99.18	EF522128.1	Yes	Nguyen; Park; Otto [68]
16.1	1097	*Citrobacter* sp*.*	99	98.63	KY630556.1	Depends on the species	Brenner et al. [34] Hasan; Sultana; Hossain [35]
16.2	1062	*Klebsiella pneumoniae*	98	99.18	AB641122.1	Yes	Boszczowski et al. [54]
16.3	1052	*Enterobacter* sp*.*	100	98.86	MW412560.1	Yes	Kus; Burrows [46]
16.4	1015	*Pseudomonas aeruginosa*	100	99.81	LR590473.1	Yes	Morello et al. [69]
16.5	1073	*Acinetobacter baumannii*	100	98.21	CP044356.1	Yes	McConnell et al. [30]
17.1	1033	*Citrobacter* sp*.*	99	99.13	MT229332.1	Depends on the species	Brenner et al. [34] Hasan; Sultana; Hossain [35]
17.2	1128	*Enterococcus* sp*.*	100	97.87	MZ229662.1	Depends on the species	Camargo et al. [42]
17.3	1047	*Acinetobacter baumannii*	100	99.24	CP042931.1	Yes	McConnell et al. [30]
17.4	1082	*Klebsiella pneumoniae*	100	99.08	CP034420.1	Yes	Boszczowski et al. [54]
17.5	–	Yeast **	–	–	–	Depends on the species	Moyad [44] Hafed et al. [45]
18.1	1119	*Acinetobacter baumannii*	100	98.75	CP045541.1	Yes	McConnell et al. [30]
18.2	1081	*Enterococcus faecalis*	100	99.35	CP045918.1	Yes	Poulsen et al. [32]
18.3	484	*Stenotrophomonas maltophilia*	100	99.17	CP040440.1	Yes	Almeida et al. [70]
18.4	995	*Citrobacter* sp*.*	100	99.90	MT229332.1	Depends on the species	Brenner et al. [34] Hasan; Sultana; Hossain [35]
18.5	–	Yeast **	–	–	–	Depends on the species	Moyad [44] Hafed et al. [45]
18.6	1033	*Comamonas* sp*.*	100	99.52	MT765012.1	No	Ghanbarinia; Kheirbadi; Mollania [72]
C1		*Azospirillum brasilense*	100	100	SAMN08346097	No	Hungria et al. [71]
C1		*A. brasilense*	100	100	SAMN08354664	No	Hungria et al. [71]
C2		*Bradyrhizobim japonicum*	100	100	AF234888	No	Menna et al. [20]
C2		*B. diazoefficiens*	100	100	AF234889	No	Menna et al. [20]
C3		*B. elkanii*	100	100	AF234890	No	Menna et al. [20]
C3		*B. elkanii*	100	100	AF237422	No	Menna et al. [20]

^*^DNA fragment (bp) sequenced; Coverage: percentage of the sequence of interest aligned with a sequence deposited at GenBank; identity: maximum identity obtained with the highest alignment scores

^**^The isolates identified as “yeast” under microscope observation were not subjected to molecular identification

^***^The isolates were numbered using the sample numbering as received in the laboratory followed by the number of the isolated colony. For example, isolate 1.5 is the 5th isolate of the sample 1

Commercial inoculants: C1, *Azospirillum brasilnse* (strains Ab-V5 and Ab-V6); C2, *Bradyrhizobium japonicum* (SEMIA5079) and *B. diazoefficiens* (SEMIA5080); C3, *B. elkanii* (SEMIA587 and SEMIA5019), respectively

Among the 84 bacterial isolates, 41 had similarity with species or genera containing at least one species reported as potentially pathogenic to humans (49%): *Enterococcus* (10), *Acinetobacter* (seven), *Citrobacter* (six), *Klebsiella* (three), *Stenotrophomonas* (three), *Enterobacter* (three), *Burkholderia* (two), *Atlantibacter* (one), *Bacillus* (one), *Escherichia* (one), *Kocuria* (one), *Paenibacillus* (one), *Pseudomonas* (one), and *Staphylococcus* (one) (Table 2).

### Metagenome analysis

**Fig. 2:**
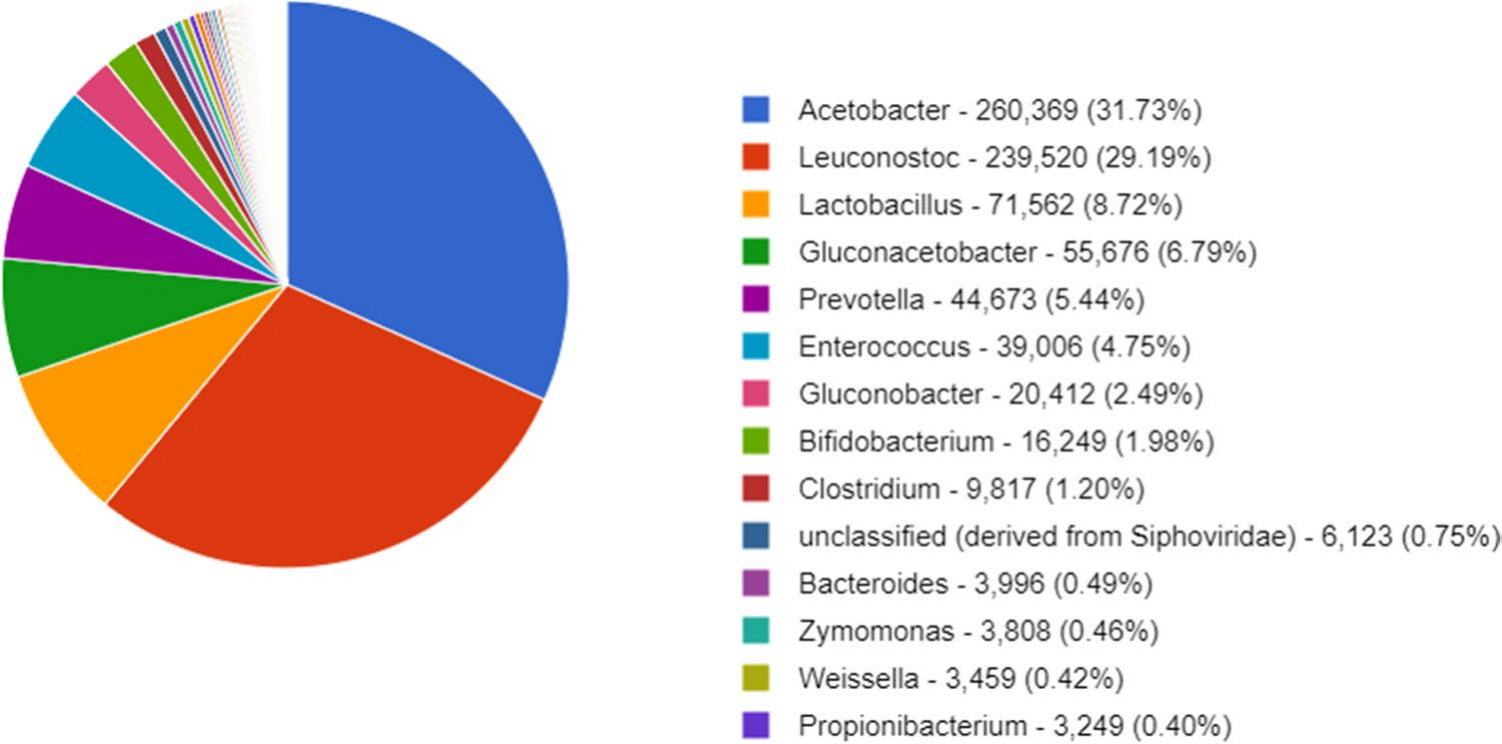
Occurrence of prevailing genera based on metagenome analysis performed with the sample no. 10 produced on farm in Palotina, Paraná, Brazil. *Azospirillum*, the target microorganism, was not detected in the sample

The shotgun approach of the sample no. 10 revealed a total of 2,467,209 sequences. After removal of the low-quality sequences and artificial duplicate reads, a total of 679,917,634 bp with average length of 276 bp was obtained. The rarefaction curve indicated that the number of sequences submitted was capable of detecting the existing diversity in the sample (not shown). Among the good-quality sequences, 1% contained ribosomal RNA genes, 90.68% encoded for proteins with known functions, and 8.14% proteins with unknown functions. Considering the automatic annotation in the MG-RAST v.4.0.4 server, the taxonomic classification of all shotgun sequences indicated that 99.23% belonged to the domain Bacteria, 0.2% to Eukaryota, 0.01% to Archaea, and 0.56% to Viruses (not shown). Among the 14 predominating genera identified in the sample, *Acetobacter* and *Leuconostoc* represented more than 50% of the sequences in the microbiome, whereas *Azospirillum*, the target microorganism in that sample, was not found (Fig. 2).

### Susceptibility to antimicrobials

The test of susceptibility to antimicrobials was carried out according to [73–75] only with 36 isolates considered of clinical relevance. Considering the CLSI protocol, 12 isolates presented no resistance to at least one antibiotic; six presented intrinsic resistance to at least one antibiotic; and 18 isolates presented single or multiple resistance (Table 3). Noteworthy, some isolates showed multiresistance to antibiotics, e.g., isolates 1.5 and 2.4, which showed high 16S rRNA gene homology with *Enterococcus faecalis*, and showed resistance to all and to five tested antibiotics, respectively.

**Table 3:** Antimicrobial resistance test applied to isolates with pathogenic potential to humans obtained from samples of inoculants produced on farm aiming the multiplication of *Bradyrhizobium* spp. and *Azospirillum brasilense* in the 2019/20 growth season

Sample Likely species/genus	Resistant to	Susceptible to
1.1 *Citrobacter* sp.	FOX, AMC, AMP^*^	GEN, AMI, CPM, CFZ, CTR, CIP, SXT, IMI, AZT, CAZ, CHL, TET
1.2 *Enterobacter* sp.	AMP, FOX, AMC	CFZ, GEN, AMI, CPM, CFZ, CTR, CIP, SXT, IMI, AZT, CAZ, CHL, TET
1.3 *Acinetobacter baumannii*	CTX, SXT	PTZ, CIP, CAZ, IMI, LEV, CPM, GEN, SAM, MRP, AMI
1.5 *Enterococcus faecalis*	GEN, LNZ, AMP, STR, VAN, PEN	
2.2 *Citrobacter* sp*.*	IMI, AZT, AMP*, CFZ*, AMC**	GEN, AMI, CPM, FOX, CTR, CIP, SXT, CAZ, CHL, TET
2.3 *Escherichia coli*	–	ERT, MRP, CIP, FOX, IMI, SXT, CTX, AMP, GEN, TET, AMC
2.4 *Enterococcus faecalis*	LNZ, AMP, STR, VAN, PEN	GEN
3.4 *Enterococcus* sp.	LNZ, PEN	AMP, GEN, STR, VAN
4.2 *Acinetobacter nosocomialis*	CTR**	SAM, CAZ, CIP, LEV, IMI, MRP, GEN, AMI, PTZ, CPM, SXT
4.4 *Enterobacter* sp.	AMI, AMC, CHL, SXT, AMP*, CFZ* FOX*, GEN**, CPM**	AZT, CAZ, CIP, IMI, TET
4.7 *Stenotrophomonas maltophilia*	–	LEV, SXT
7.1 *Citrobacter* sp.	AMI, AZT, GEN, IMI, AMP*, CFZ*, CTR**	AMC, CPM, FOX, CIP, SXT, CAZ, CHL, TET
7.3 *Klebsiella pneumoniae*	AMP*	CFZ, GEN, AMI, AMC, CPM, FOX, CTR, CIP, SXT, IMI, AZT, CAZ, CHL, TET
7.4 *Stenotrophomonas* sp.	–	LEV, SXT
7.5 *Atlantibacter hermannii*	AMP	CFZ, GEN, AMI, AMC, CPM, FOX, CTR, CIP, SXT, IMI, AZT, CAZ, CHL, TET
8.4 *Enterococcus faecalis*	–	AMP, LNZ, PEN, STR, VAN
10.1 *Enterococcus faecalis*	STR	AMP, LNZ, PEN, VAN
11.3 *Enterococcus hirae*	–	AMP, LNZ, PEN, STR, VAN
12.1 *Acinetobacter* sp.	–	CAZ, SXT, CIP, IMI, LEV, MRP,PTZ, CTR, AMI, SUL, CPM, GEN
14.1 *Enterococcus faecalis*	–	AMP, LNZ, PEN, STR, VAN
15.2 *Staphylococcus epidermidis*	ERY	GEN, CLI, CIP, OXA, TET, CHL, LNZ, PEN
16.1 *Citrobacter* sp.	CFZ*, AMC*	SXT, AMI, CHL, TET, IMI, AMP, CIP, CAZ, CFZ, GEN, CTR, AZT, CPM, AMC
16.2 *Klebsiella pneumoniae*	AMP*	AMC, SXT, AMI, CHL, TET, IMI, CFZ, CIP, CAZ, CFZ, GEN, CTR, AZT, CPM, AMC
16.3 *Enterobacter* sp.	AMC*, AMP*, CFZ*	SXT, CHL, TET, IMI, CFZ, CIP, CAZ, GEN, CTR, AZT, CPM
16.4 *Pseudomonas aeruginosa*	–	GEN, CFZ, LEV, MRP, IMI, CPM,CIP, AZT, PTZ
16.5 *Acinetobacter baumannii*	CTR	CAZ, SXT, CIP, IMI, LEV, MRP,PTZ, AMI, SUL, CPM, GEN
17.1 *Citrobacter* sp.	AMP*, CFZ*, AMC*	AMC, SXT, AMI, CHL, TET, IMI, CIP, CAZ, CFZ, GEN, CTR, AZT, CPM
17.2 *Enterococcus* sp.	–	GEN, LNZ, AMP, STR, VAN, PEN
17.3 *Acinetobacter baumannii*	PTZ	CAZ, SXT, CIP, IMI, LEV, MRP,CTR, AMI, SUL, CPM, GEN
17.4 *Klebsiella pneumoniae*	AMP*	AMC, SXT, AMI, CHL, TET, IMI, CFZ, CIP, CAZ, CFZ, GEN, CTR, AZT, CPM, AMC
18.1 *Acinetobacter baumannii*	–	CAZ, SXT, CIP, IMI, LEV, MRP,CTR, AMI, SUL, CPM, GEN, PTZ
18.2 *Enterococcus faecalis*	–	AMP, LNZ, PEN, STR, VAN
18.3 *Stenotrophomonas maltophilia*	–	LEV, SXT
18.4 *Citrobacter* sp.	AMI, CTR, AZT, AMP*, AMC*, CFZ*, GEN**, IMI**	SXT, CHL, TET, CIP, CAZ, CPM

^*^Intrinsic resistance (natural of the microorganism)

^**^Intermediate resistance

*AMI* amikacin, *AMC* amoxicillin + clavulanate, *AMP* ampicillin, *SAM* ampicillin + sulbactam, *AZT* aztreonam, *CFZ* cefazolin, *CPM* cefepime, *CTX* cefotaxime, *FOX* cefoxitin, *CAZ* ceftazidime, *CTR* ceftriaxone, *CIP* ciprofloxacin, *CLI* clindamycin, *CHL* chloramphenicol, *ERY* erythromycin, *ERT* ertapenem, *GEN* gentamycin, *IMI* imipenem, *LNZ* linezolid, *LEV* levofloxacin, *MRP* meropenem, *PEN* penicillin, *PTZ* piperacillin + tazobactam, *STR* streptomycin, *SUL* sulbactam, *SXT* sulfamethoxazole + trimetropim, *TET* tetracycline, *VAN* vancomycin

## Discussion

Among 84 isolates, 25 genera were identified, 44% of which are known to harbor potential human pathogens, whereas only one isolate (5.2) showed 16S rRNA gene homology with the target microorganism *A. brasilense*. That was a case in which the sample was taken only 4 h after the tank had been inoculated with a commercial inoculant. Thus, the isolate probably originated from the commercial inoculant used as inoculum, not from the multiplication, since the short time between the addition of inoculum and the sampling may still have allowed the microorganism to survive. No other sample provided colonies identified as *Azospirillum*, showing that the target microorganism is eliminated or suppressed as the growth media become dominated by contaminating microorganisms. In addition, among the six samples aiming to multiply *Bradyrhizobium*, no isolate corresponded to the target bacteria.

Multiplication of microorganisms must assure several minimal microbiological procedures to guarantee that the target microorganism prevails in the culture medium. In the case of *Azospirillum* and mainly *Bradyrhizobium*, a slow-growing bacterium [15], several other microbial contaminants dominate the culture medium as they have shorter generation times, i.e., higher growth rates than the target bacteria. In many cases, the carbon source in the culture medium used for on farm production is not appropriate. For example, the use of sucrose provided as molasses for growth of *Bradyrhizobium* is not appropriate, as the preferred carbon sources are glycerol or mannitol [15]. Besides competition with contaminating microorganisms, the physical–chemical characteristics in the culture medium are also inappropriate for growth of the target microorganisms. For example, the adequate range of pH for *Bradyrhizobium* and *Azospirillum* is between 6.8 and 7.0 [15, 16, 76]; however, 94.4% of the samples had pH ranging from 3.6 to 5.9. The low pH can also favor the growth of contaminating microorganisms adapted to low pH and thus contributing to suppress the target microorganisms.

The lack of standardization in the incubation time is another problem in the samples taken from on farm production in this study. The average growth time of the recommended *Bradyrhizobium* strains to reach the ideal concentration (at least 1 × 10^9^ cells mL^−1^) in the inoculant is approximately 7 days [76–79]. Similarly, *A. brasilense* has a growth time of about 5 days to reach at least 1 × 10^8^ cells mL^−1^ [80]. In contrast, many contaminants have much shorter generation times, and dominate the culture medium in less than 24 h. Contaminating microorganisms compete for resources in the growth medium that becomes nutritionally poor and can also release inhibiting byproducts [81]. Therefore, it is reasonable to conclude that the high multiplication rates of the contaminating microorganisms, in addition to the low growth rates of the target microorganisms, result in the rapid depletion of the culture medium and enrichment with metabolites that inhibit the development of slow-growing microorganisms, like *Bradyrhizobium* and *Azospirillum*.

Multiplication of microorganisms without strict quality control can be risky to humans, animals, crops, and environment. Many contaminants are potentially pathogenic to humans and may cause various diseases, posing risks to the health of individuals who handle these products, or even final consumers if applied to products consumed *in natura*. Although potentially pathogenic microorganisms are found in the environment, they usually do not cause risk due to the low potential of inoculum in the environment. However, the multiplication of this microbial population in contaminated culture media could also magnify risks of infections or contaminations. For example, microorganisms from genera like *Enterococcus*, for which similar sequences were found in 61.1% of the samples, are frequently related to bacteremia, septicemia, urinary tract infections, abscesses, meningitis, and endocarditis [32, 82–84]. Some isolates also presented high genetic similarity with *Citrobacter freundii* [85], *Enterobacter cloacae* [86, 87], and *Paenibacillus polymyxa* [88], which are also potentially pathogenic to plants [86–88].

The possibility to carry genes of resistance to antimicrobials is a further concern in magnifying the population of potentially pathogenic contaminants in the on farm production. The spread of such genes in the environment may restrict the resources to fight infections. Some opportunist pathogens like *Stenotrophomonas maltophilia* are intrinsically resistant to several antimicrobials and collaborate to spread genes of resistance in the environment [70]. In this study, 12 isolates presented non-intrinsic resistance to antimicrobials, and 10 isolates presented resistance to two or more antimicrobials (1.1, 1.2, 1.3, 1.5, 2.2, 2.4, 3.4, 4.4, 7.1, and 18.4), what is an additional concerning issue.

Isolates identified microscopically as yeasts were not sequenced for genetic comparisons with sequences deposited in ribosomal databanks. However, some genera of yeasts can also cause injuries to humans and animals. Although yeasts are used in the manufacture of breads and beer, without any risk to humans and animals, like *Saccharomyces cerevisiae*, the genus *Candida* is the main pathogenic yeast and comprises approximately 200 species [89].

The approach based on metagenome for sample no. 10 showed that only contaminating microorganisms prevailed in the on farm sample. Although four morphologically distinct colonies were isolated from that sample based on the culture medium approach, the metagenome approach revealed more than 10 genera, including the ones isolated based on the cultivation method. This indicates that the amount of contaminating microorganisms in the on-farm multiplications can be far higher than revealed by the culture-based method. In addition, even using a more sensitive method, the target microorganism was not found in that sample.

Studies on inoculants produced on farm and their impacts on production systems and potential risks to public health are scarce. However, our findings corroborate previous studies on bioinsecticides produced on farm, which revealed low concentration or absence of the target microorganisms *Bacillus thuringiensis* [12], and absence of *Chromobacterium subtsugae* and *Saccharopolyspora spinosa* [13]. However, there was high prevalence of contaminants in the samples, some of them potentially pathogenic to humans [12, 13].

The negative effect of low-quality bioproducts produced on farm goes beyond the risk to Brazilian quality of agricultural products, crops, and environment, because the benefits to the crops cannot be reached with its use. The lack of effect for not containing the target microorganism might put in doubt consolidated technologies that are important to the sustainability of cropping systems like the BNF in soybean by inoculation with *Bradyrhizobium* [3, 4], and more recently inoculation of grasses and co-inoculation of soybean with *Azospirillum* [7, 8, 11].

In conclusion, the samples of inoculants produced on farm assessed in this study were highly contaminated with several non-target microorganisms, whereas the target microorganisms *Azospirillum* and *Bradyrhizobium* were not detected in the great majority of the samples. In addition, the occurrence of contaminants presenting high genetic similarity with potentially pathogenic microorganisms, some of them carrying non-intrinsic resistance or multiresistance to antimicrobials, may indicate risk to human health.
